# Global research trends in *Fusobacterium nucleatum* and colorectal cancer: a bibliometric and visual analysis

**DOI:** 10.3389/fcimb.2026.1851307

**Published:** 2026-09-03

**Authors:** Guanhua Lyu, Yingge Yue, Manfei Duan, Junhao Chen, Yanyan Chen, Junyu Liu, Xiangyu Wang

**Affiliations:** Shanxi Medical University School and Hospital of Stomatology, Shanxi Province Key Laboratory of Oral Diseases Prevention and New Materials, Taiyuan, China

**Keywords:** *Fusobacterium nucleatum*, colorectal cancer, CiteSpace, VOSviewer, bibliometrics, visualization

## Abstract

**Background:**

*Fusobacterium nucleatum* (*F. nucleatum*) is increasingly recognized as a key pathobiont in colorectal cancer (CRC), driving tumor progression through immune evasion, inflammation, and metabolic reprogramming. Nevertheless, a comprehensive bibliometric analysis of the literature on *F. nucleatum* and CRC remains a gap in the current research landscape. Here, we integrate bibliometric trend analysis with a narrative synthesis of key mechanistic insights. Unlike traditional narrative reviews, this study systematically quantifies global research trends, collaboration networks, and thematic evolution using integrated bibliometric tools (CiteSpace, VOSviewer, and Bibliometrix).

**Methods:**

This study employed bibliometric analysis to explore the current status of research related to *F. nucleatum* and CRC. Publications from 2000 to 2025 were retrieved from the Web of Science Core Collection (WOSCC) and Scopus. We used VOSviewer, CiteSpace and Bibliometrix to visualize countries, institutions, authors, keywords, journals and references. Statistical analysis was conducted using Microsoft Office Excel.

**Results:**

The annual number of publications and their relative percentages concerning *F. nucleatum* and CRC exhibited a steady upward trend from 2009 to 2025, with China (n=708), the United States (n=584), and Italy(n=119) dominating the field. Harvard University and Shanghai Jiao Tong University emerged as leading institutions, while Yu Jun and Shuji Ogino were the most prolific authors. Keyword analysis identified 7 clusters. The most popular journal in this field is Gut. Limitations include potential selection bias from using only two databases (WOSCC and Scopus) and restriction to English-language publications.

**Conclusion:**

Our research provides a comprehensive overview of the research trends and key focal areas in the study of *F. nucleatum* and CRC. The analysis results show that the annual publication volume in this field has steadily increased, indicating that researchers’ attention to this topic has grown continuously. Future research directions suggested by the literature include further exploration of *F. nucleatum*-targeted therapies, biomarker validation, and multi-omics approaches, although these remain at an early stage.

## 1 Introduction

Colorectal cancer (CRC) is the third most diagnosed malignancy and the second leading cause of cancer-related death worldwide. It imposes a staggering burden on public health systems, with approximately 1.9 million new cases and 900,000 deaths annually (Sung et al., 2021; Xi and Xu, 2021). Advancements in screening and treatment have improved survival rates in high-income countries. However, significant disparities persist in low- and middle-income regions. Late-stage diagnoses and limited access to therapies in these regions further increase mortality rates (Arnold et al., 2017; Douaiher et al., 2017). CRC has a multifactorial etiology. It involves complex interactions between genetic predisposition and modifiable risk factors, including obesity, sedentary lifestyles and smoking (Brenner and Chen, 2018). Despite these established associations, emerging evidence underscores the pivotal role of gut microbiota dysbiosis as both a biomarker and active contributor to CRC pathogenesis (Garrett, 2019; Wirbel et al., 2019).

The relationship between microorganisms and cancer has long been a significant research focus. Accumulating evidence demonstrates that bacteria, viruses, and fungi influence cancer development and treatment response through diverse mechanisms (Plummer et al., 2016). Both gut and tissue-specific microbiota play critical roles in cancer pathogenesis and therapeutic outcomes. Some bacteria can activate the immune system, thereby enhancing anti-tumor immune responses, while others may suppress immune responses and promote tumor growth (Hanahan, 2022). The International Agency for Research on Cancer (IARC) has classified 11 microorganisms as direct carcinogens, such as *Helicobacter pylori* and high-risk human papillomavirus (HPV). However, a greater number of microorganisms act as “accomplices” in cancer development. For instance, gut microbiota dysbiosis can suppress antitumor immunosurveillance, such as by reducing secondary bile acids that inhibit the aggregation of natural killer T cells in the liver (Sepich-Poore et al., 2021).

The human gut microbiome, a dynamic ecosystem comprising trillions of microorganisms, regulates host immunity, metabolism, and epithelial integrity (Rinninella et al., 2019). Among these microbial players, *Fusobacterium nucleatum* (*F. nucleatum*), a Gram-negative anaerobe predominantly colonizing the oral cavity, has emerged as a tumor-specific pathobiont with profound oncogenic potential (Kostic et al., 2013; Brennan and Garrett, 2019). Its enrichment in CRC tissues correlates with advanced tumor stages, chemotherapy resistance, and poor prognosis, positioning it as a critical therapeutic target (Gur et al., 2015; Mima et al., 2015).

Recent clinical studies further highlight its translational relevance. For instance, *F. nucleatum*-positive CRC tumors exhibit resistance to 5-fluorouracil and oxaliplatin, while combinatorial approaches targeting microbial pathways (e.g., metronidazole with PD-1 inhibitors) show promise in restoring therapeutic efficacy (Yu et al., 2017; Hamada et al., 2018). Despite these strides, the intellectual landscape of *F. nucleatum*-CRC research remains fragmented, with limited synthesis of thematic evolution, collaborative networks, and emerging frontiers.

Bibliometric analysis, a quantitative methodology for mapping scientific knowledge domains, offers a powerful tool to delineate research trends, identify knowledge gaps, and forecast innovations (Koskinen et al., 2008). Although several narrative reviews have summarized the biological functions and pathogenic mechanisms of *F. nucleatum* in CRC, a dedicated bibliometric analysis systematically evaluating the global research landscape, collaboration patterns, knowledge structure, and emerging trends in this field remains lacking (Jiang et al., 2026; Wuyi and Tao, 2026). Unlike traditional narrative reviews or meta-analyses, it can identify influential papers, collaboration networks, and evolving research fronts without requiring prior hypothesis formulation. Applying this approach to *F. nucleatum*-CRC research is particularly timely given the exponential growth of publications in this area over the past decade. Here, we utilized VOSviewer, CiteSpace and Bibliometrix to analyze 2010 papers from the Web of Science Core Collection (WOSCC) and Scopus database, and drew maps of research trends, key collaboration networks, and hot topics such as tumor microenvironment and immunotherapy. This study has two complementary objectives: (1) to quantitatively delineate the research landscape, collaboration networks, and emerging hotspots using bibliometric tools; and (2) to qualitatively synthesize the established molecular mechanisms of *F. nucleatum* in colorectal carcinogenesis, based on the most highly cited and burst references identified from the bibliometric analysis. This study offers a structured overview that may help guide future research priorities, including basic, translational, and clinical investigations.

## 2 Methods

### 2.1 Data retrieval and collection strategy

A systematic bibliometric analysis was conducted using WOSCC and Scopus database, renowned for their comprehensive coverage of high-impact scientific literature and compatibility with bibliometric tools (Falagas et al., 2008). The search formula of WOSCC is: TS= (“*Fusobacterium nucleatum*” OR “*F. nucleatum*”) AND (“colorectal cancer” OR “colon cancer” OR “rectal cancer” OR “colorectal carcinoma” OR “colorectal tumor” OR “colorectal neoplasm”). The search formula of Scopus is: TITLE-ABS-KEY ((“*Fusobacterium nucleatum*” OR “*F. nucleatum*”) AND (“colorectal cancer” OR “colon cancer” OR “rectal cancer” OR “colorectal carcinoma” OR “colorectal tumor” OR “colorectal neoplasm”)).

**Figure 1:**
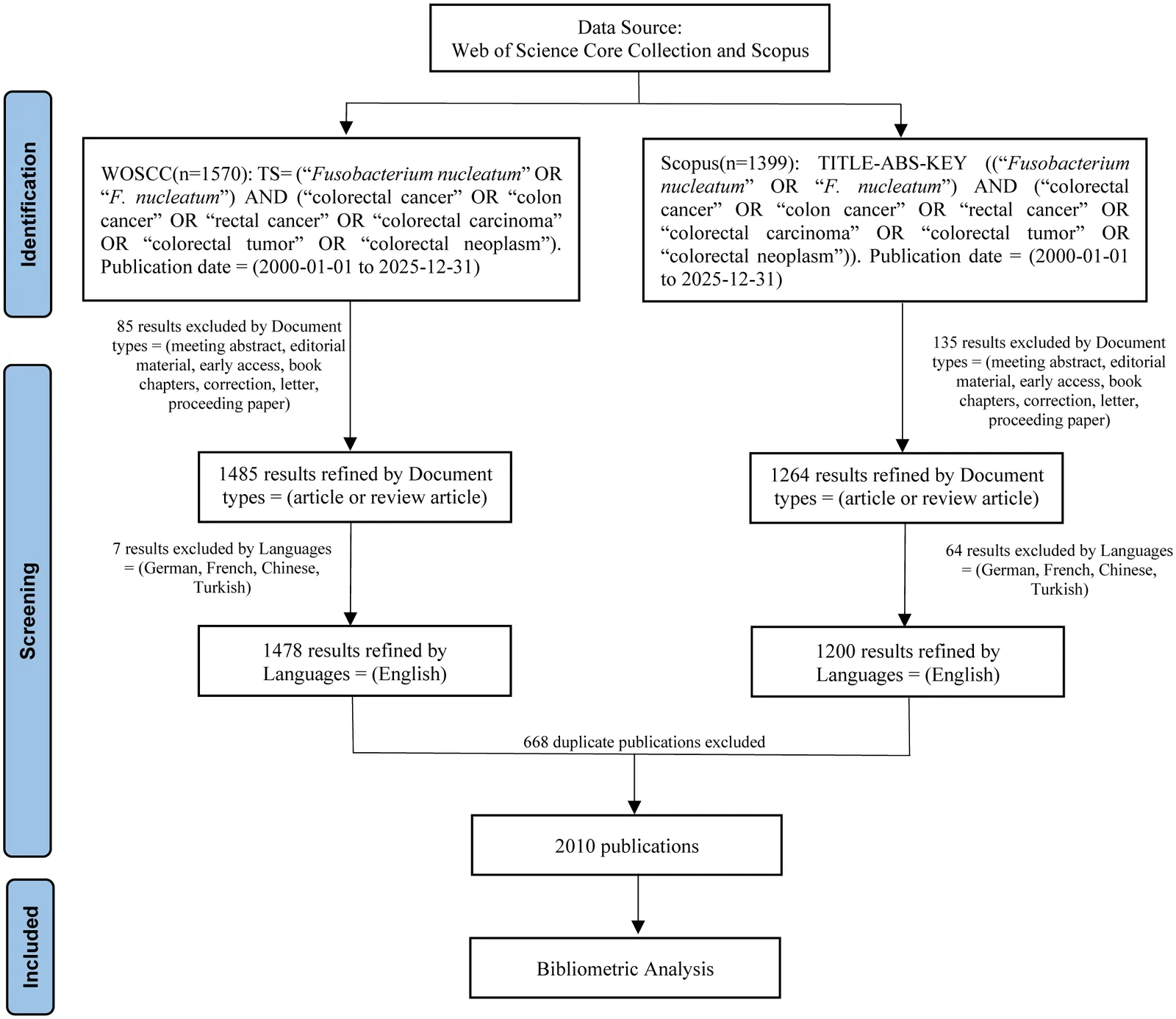
Flowchart of literature selection based on the PRISMA guidelines.

The study selection process was conducted in accordance with the PRISMA guidelines, and the inclusion and exclusion criteria are summarized in the PRISMA flowchart (**Figure 1**). The criteria for screening and inclusion were: (1) data source as Web of Science Core Collection (SCI-EXPANDED, SSCI) and Scopus. (2) publication type as “article” or “review”, (3) language as “English”, and (4) published between 2000-01–01 and 2025-12-31. Duplicate records across WOSCC and Scopus databases were removed using NoteExpress. To combine the literature from WOSCC and Scopus and remove duplicates, we imported WOSCC records into NoteExpress using the “Web of Science New” filter, and imported Scopus records (exported in RIS format) using the “RefMan - (RIS)” filter. The “Duplicate Check” function in NoteExpress was then applied with the “Title” field as the matching criterion to delete exact duplicate records from the library. After automatic deduplication, we independently performed a manual review of all remaining records to identify any missed duplicates.

**Figure 2:**
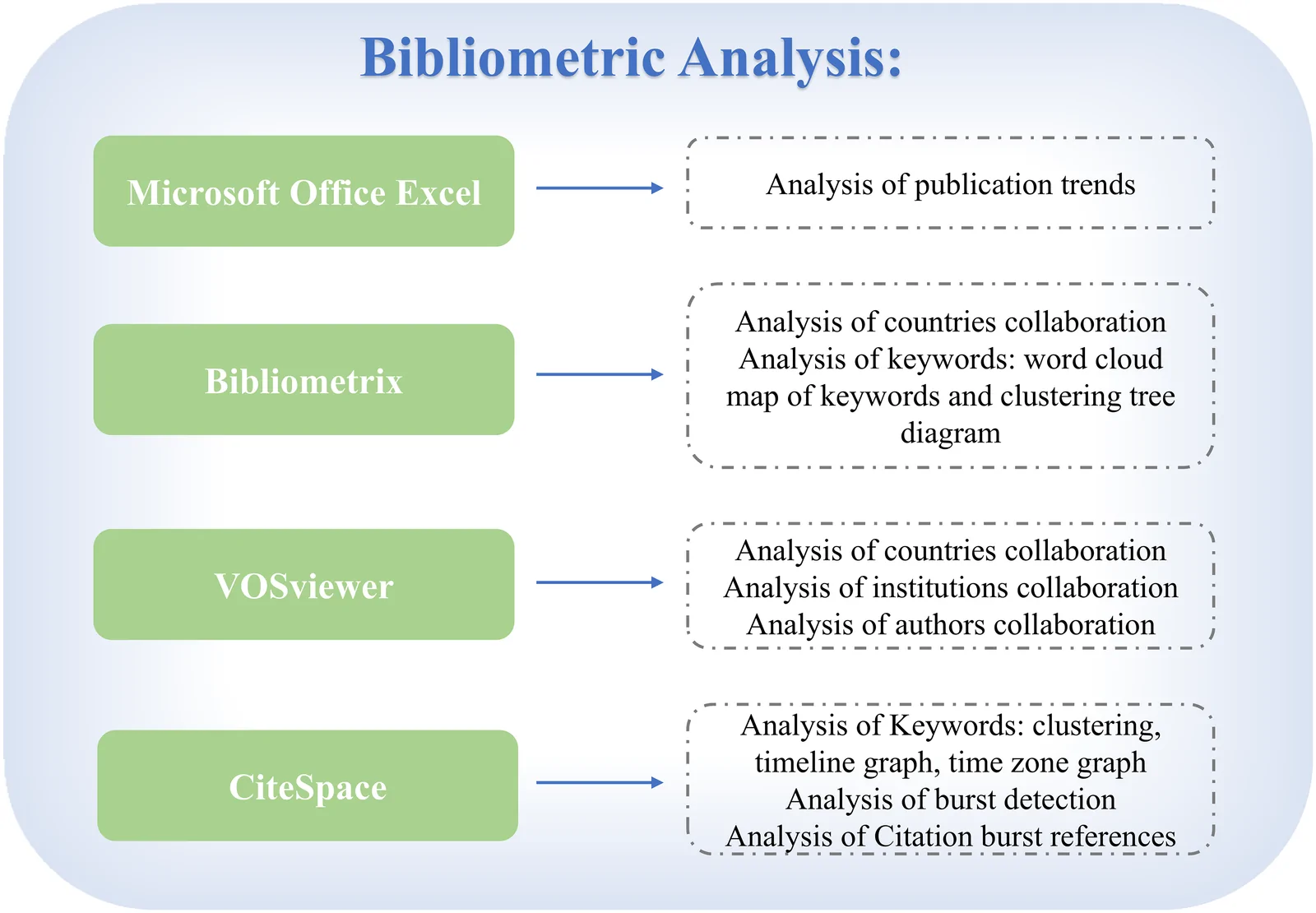
Flowchart of the application process of bibliometric analysis method.

All searches were completed and data downloaded on the same day, which was done to minimize errors that might be caused by routine database updates. After applying the inclusion/exclusion criteria and removing duplicates, a total of 2010 unique publications were retained for bibliometric analysis. The flowchart of the application of bibliometric analysis methods is shown in **Figure 2**. Since all data were obtained from public databases without involving human subjects or private information, ethics committee approval was not required.

### 2.2 Data analysis

In VOSviewer (version 1.6.20), 2010 documents that meet the inclusion exclusion criteria need to be exported in the format of Tab delimited file, with Record Content as “author, title, source, abstract”. In the study of this paper, the software performs country, institution and co-occurrence analysis of authors. 2010 documents that meet the inclusion exclusion criteria in CiteSpace (version 6.4.R1) need to be exported in RefWorks format and saved as “download_***.txt”. The final export was “fully documented and cited references”. In this study, the software was used to perform keyword co-occurrence analysis, keyword clustering analysis, timeline view, time zone view, keyword burst analysis, analysis of cited journals and cited references. CiteSpace used the following settings: link retention factor (LRF = 2.5), e top N (e = 1), period (2000-2024), slice length =1, year of review (LBY = 5), link (strength: cosine, range: in slice), selection criteria (g index: k = 2). The g-index is a statistical indicator used to extract key literature in CiteSpace. The g-index was selected as the node selection criterion because it considers both the productivity and citation impact of publications and provides a more flexible and adaptive selection strategy than the fixed Top N approach. Furthermore, the scaling factor k allows control of the network size while preserving influential nodes. The proportional parameter k scales the g-index value to determine network size. We selected k=2 based on comprehensive considerations: k=2 yielded the highest clustering quality metrics (Modularity Q = 0.4606 > 0.3, Silhouette S = 0.7882 > 0.5), indicating statistically significant and consistent clustering results. k=2 denotes that the top 2 literature ranked by g-index is selected as key nodes. By adjusting the proportional parameter k, the size of the network can be adjusted. The larger k is, the larger the network is (Zhao et al., 2025). We adopt this setting to identify literature with genuine structural importance while ensuring the readability of the network. Microsoft Office Excel for data management and publication output analysis. Bibliometric analysis was conducted using the Bibliometrix tool. This online analysis platform facilitates the display of national cooperation on the map, determines the most cited keywords, and generates keyword clouds and tree diagrams.

In CiteSpace, the size of a node can reflect the number of times the node is cited or occurs (Yao et al., 2022). Nodes with centrality exceeding 0.1 are surrounded by a purple ring, marking them as pivot points, which are usually considered hotspots or key nodes in their respective fields (Liu et al., 2022b). Links connecting nodes illustrate the association between two nodes, and an increase in the number of lines indicates the growth of collaboration, while the thickness of the connecting lines in the keyword co-occurrence network is a visual indicator of the closeness of the relationship between keywords (Wu et al., 2023). Besides, different colors correspond to different years and can reflect the relationship of time. In addition to bibliometric mapping, we conducted a narrative review of the molecular mechanisms by which *F. nucleatum* promotes colorectal cancer.

## 3 Results

### 3.1 Global trend in publication outputs

**Figure 3:**
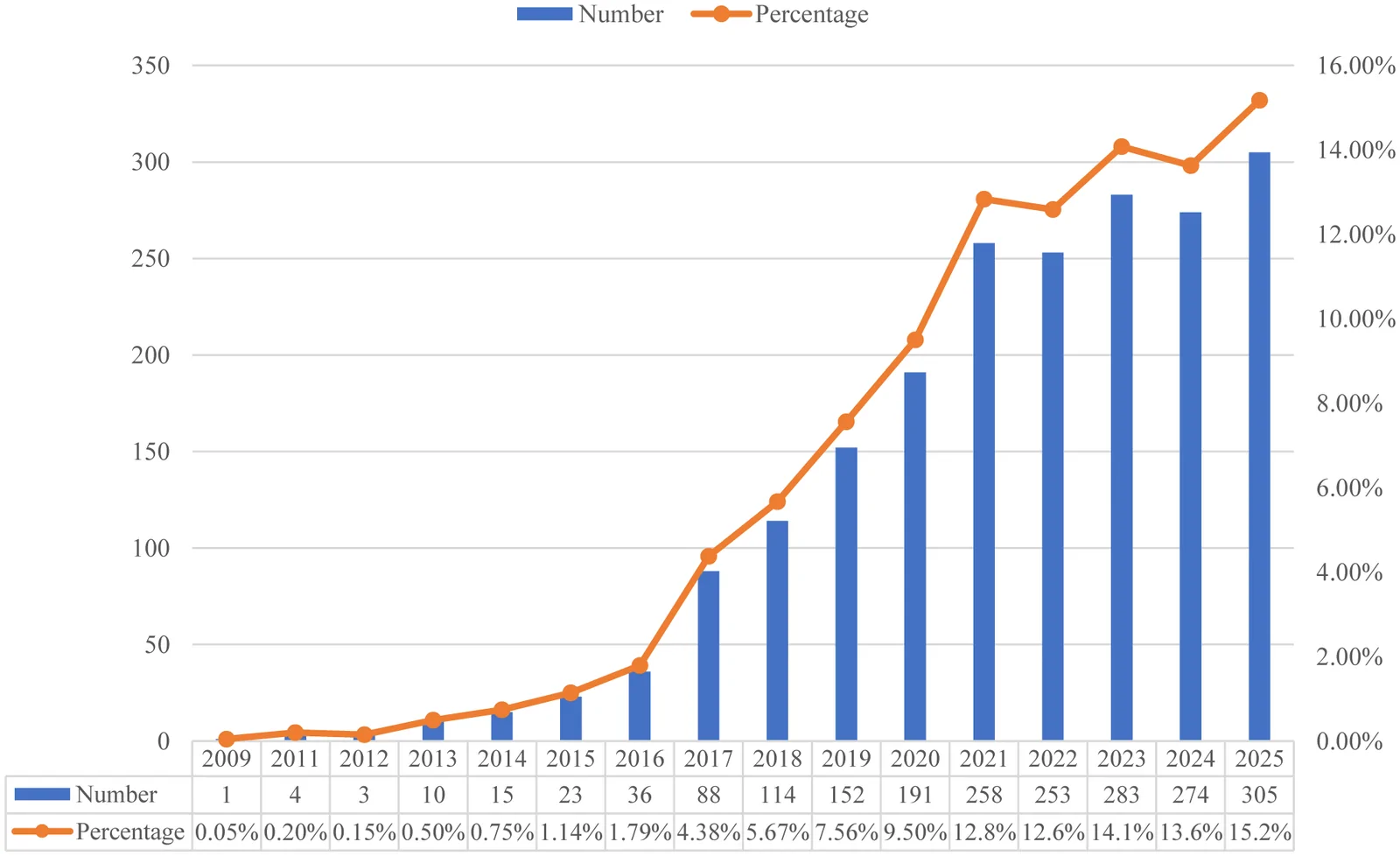
Worldwide trend in publications of *F. nucleatum* and CRC. The annual publication count remained low before 2016, then entered a rapid growth phase (2017-2021), and stabilized at a high level thereafter (2021–2025), indicating sustained research interest.

A total of 2010 publications on *F. nucleatum* and CRC were identified from WOSCC and Scopus (2000-2025). As illustrated in **Figure 3**, the annual number of publications and their relative percentages concerning *F. nucleatum* and CRC exhibited a steady upward trend from 2009 to 2025. The developmental trajectory of this research field can be broadly divided into three distinct phases. During the initial exploration phase (2009–2016), the annual publication volume was relatively low, with a gradual increase to 36 papers (1.79%) in 2016. This reflects the nascent stage of research following the landmark discoveries of *F. nucleatum* enrichment in CRC tissues. Subsequently, the field entered a period of rapid growth (2017–2021), characterized by a sharp increase in publications from 88 (4.38%) in 2017 to 258 (12.8%) in 2021. This surge reflects growing interest in the tumor microenvironment and gut microbiota, driven by advances in sequencing technology. In the current mature phase (2021–2025), although the explosive growth rate has stabilized, the overall annual output remains at a consistently high level. Notably, the publication volume reached its peak at 305 papers (15.2%) in 2025. This trajectory mirrors the progressive understanding of *F. nucleatum* in CRC, from initial discovery to mechanistic exploration and recent translational focus. Overall, these literature trends underscore the increasingly pivotal role of *F. nucleatum* in CRC research and suggest that it continues to be a subject of intense and sustained scientific interest. The increasing publication trend was indicative of the growing importance and recognition of *F. nucleatum* and CRC, suggesting a promising future for the field. This rise in academic contributions was attributed to enhanced technological advancements, funding availability, and a greater global research collaboration. The phased growth of publications mirrors the evolving research focus of this field, and these changes will be interpreted combined with research hotspots in the discussion section.

### 3.2 Distribution of countries and institutions

**Figure 4:**
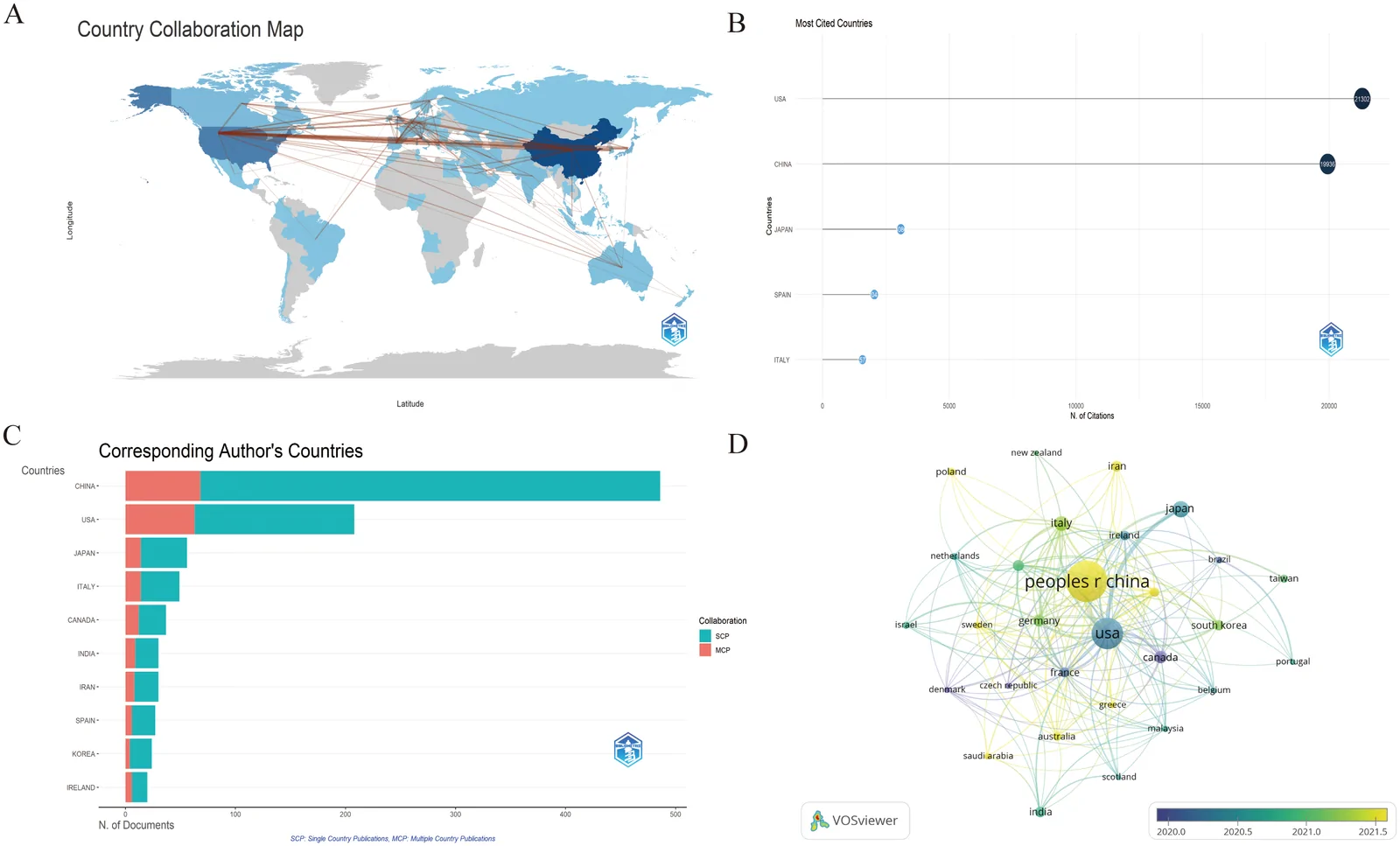
The visualization and analysis of the international collaboration networks on research of *F. nucleatum* and CRC. **(A)** Collaboration visualization of countries on the research of *F. nucleatum* and CRC. The variation in color intensity (ranging from light to dark blue) corresponds to the number of publications from each country. The thickness of the red lines connecting any two countries directly reflects the strength of their collaborative ties. **(B)** Chart of the top 5 most cited countries in the research field of *F. nucleatum* and CRC. The horizontal axis represents the total number of citations obtained by publications affiliated with each country. **(C)** The Corresponding Author’s Countries collaboration analysis of this study. SCP, single-country publication; MCP, published in multiple countries. **(D)** Countries collaboration network analysis. Each node represents a country. The size of the node represents the number of publications, and the thickness of the connection between the nodes represents the intensity of cooperation.

The 2010 publications came from 71 countries and 1675 institutions, The top 10 countries are in Asia, North America and Europe. The country with the largest number of publications is China (n= 708), followed by the United States (n= 584) and Italy (n= 119). These countries demonstrated high levels of output and a central cooperative role. In order to show the country distribution more intuitively, we generated a geographic map of the distribution of the countries (**Figure 4A**). The national cooperation network diagram, reveals the close ties among the United States, China, Germany, the United Kingdom, France, Japan, South Korea and other countries, demonstrating extensive cooperative research efforts in this field. The USA and China were the most academically influential countries in *F. nucleatum* and CRC research, with total citations of 21302 and 19936, respectively, far exceeding those of other countries (**Figure 4B**). **Figure 4C** shows that China led in single-country publications (SCP), while both China and the United States maintain extensive multi-country publication (MCP) networks that drive global research progress. Although China ranks first in publication volume, its proportion of MCP is lower than that of the United States, suggesting that Chinese research in this field remains relatively insular. Strengthening international partnerships could enhance global visibility and impact. This might be due to (1) the abundant domestic research funds, which support independent large-scale studies; (2) language barriers may have restricted communication and co-authorship with teams outside China; (3) the research culture might be more inclined toward local collaboration. China and the United States dominate global publications largely due to their abundant clinical sample resources, long-term research funding support and sophisticated experimental platforms for microbiology and oncology.

We screened 29 countries for visualization based on the number of publications at least 9, and constructed a collaborative network based on the number and relationships of publications in each country (**Figure 4D**). In VOSviewer, node size generally reflects the number of papers published in a country, with China and the United States having significantly larger nodes, reflecting their highest volume of publications. If two countries have articles in common, a line is formed between them, and the thickness of the line indicates the closer the relationship between the two elements. The thicker the line between two labels, the closer the relationship between two labels is. It is important to note that both China and the United States have many connections that indicate that they have cooperative relationships with multiple countries. In addition, we found that regional clusters included (1) Asia-Pacific: Intensive collaboration between China, Japan, and South Korea. (2) North America-Asia: Strong U.S.-China partnerships, particularly in molecular mechanism studies. (3) Europe: Germany and Italy serving as hubs for clinical-translational research.

**Figure 5:**
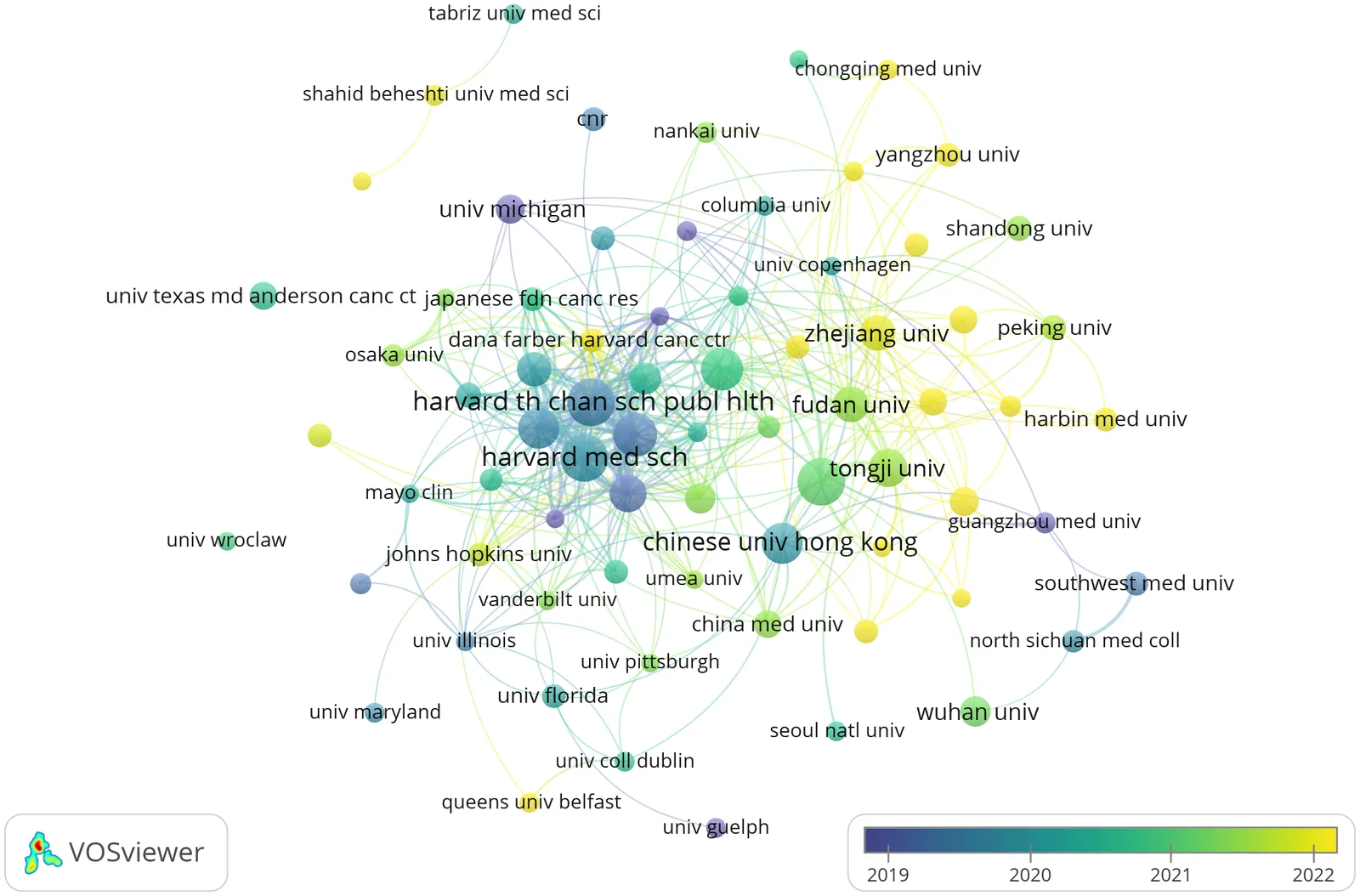
The visualization of institutions collaboration on research of *F. nucleatum* and CRC. Early institutional collaborations are represented by nodes marked in purple or blue, while current institutional collaborations are represented by nodes marked in yellow. Institutions from the USA (e.g., Harvard University) and China (e.g., Shanghai Jiao Tong University) serve as central hubs in the collaboration network, with dense connections between eastern USA and southern China.

The top 10 institutions are spread across two countries, with seven in the United States and three in China (**Table 1**). The institutions with the highest number of published papers are Harvard University (n=57) and Harvard T.H. Chan School of Public Health (n=48). The CIBER-Centro de Investigación Biomédica en Red (Spain) demonstrated the highest centrality, highlighting its interdisciplinary collaborations in microbiota-host interaction studies. Some regional collaboration clusters, such as the dense connections between institutions in the eastern United States and southern China, show the close cooperation between research institutions in these regions. We then selected 73 institutions for visualization based on a minimum number of seven publications, and constructed a collaborative network based on the number of publications and relationships for each institution (**Figure 5**). Harvard University (including Harvard Medical School and the Harvard T.H. Chan School of Public Health) and Dana-Farber Cancer Institute are core nodes in the network, indicating that these institutions play a key role in research in this area and have partnerships with multiple other institutions. Furthermore, there is extensive international collaboration among research institutions, especially between the United States, China and Europe.

**Table 1:** Top 10 countries and institutions on research of *F. nucleatum* and CRC.

Rank	Country	Number	Institution	Number
1	China (Asia)	708	Harvard University (USA)	57
2	USA (North America)	584	Harvard T.H. Chan School of Public Health (USA)	48
3	Italy (Europe)	119	Harvard Medical School (USA)	47
4	Japan (Asia)	109	Dana-Farber Cancer Institute (USA)	45
5	Canada (North America)	89	Shanghai Jiao Tong University (China)	44
6	India (Asia)	62	Massachusetts Institute of Technology (USA)	41
7	Iran (Asia)	56	Broad Institute (USA)	41
8	Germany (Europe)	55	Brigham & Women’s Hospital (USA)	39
9	Spain (Europe)	50	Sun Yat Sen University (China)	34
10	South Korea (Asia)	49	Chinese University of Hong Kong (China)	33

### 3.3 Authors and co-cited authors

**Figure 6:**
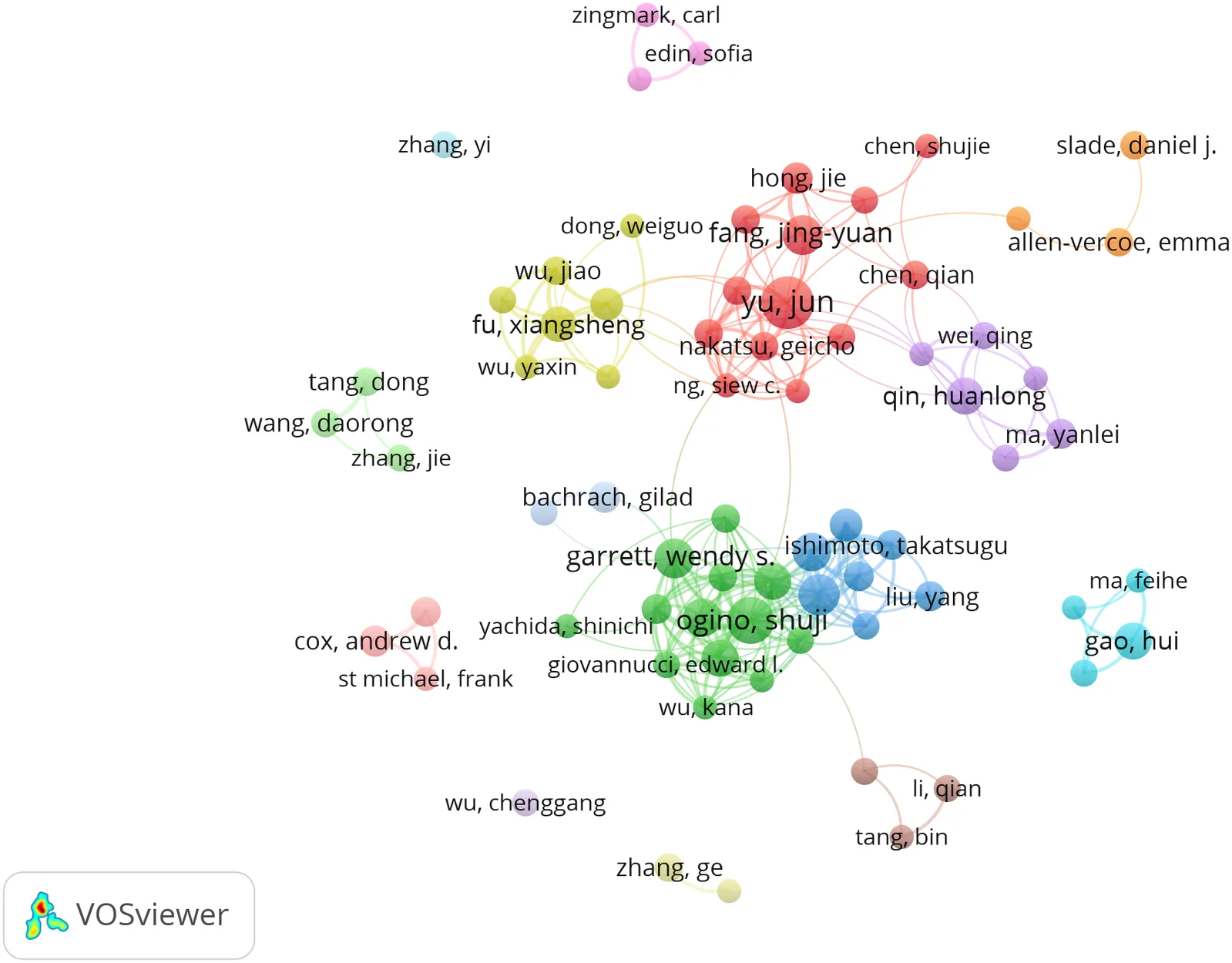
The visualization of authors. Nodes represent authors; size indicates publication count (larger = more publications); color denotes cluster affiliation; line thickness reflects collaboration frequency.

There were 6,856 authors in the study, and we counted the top five authors and co-cited authors (**Table 2**). Yu Jun of China was the most prolific author with 27 papers, followed by Shuji Ogino of the United States with 22 papers. Professor Yu Jun’s team has made remarkable achievements in the study of the relationship between *F. nucleatum* and CRC. The study shows the potential positive role of *F. nucleatum* in the treatment of microsatellite stable colorectal cancer (MSS CRC), providing a new strategy for immunotherapy. The team’s research, published in the internationally renowned journal Cancer Cell, reveals the potential positive role of gut bacteria *F. nucleatum* in the treatment of MSS CRC (Wang et al., 2025). Professor Ogino Shuji has made remarkable achievements in his research on the relationship between *F. nucleatum* and CRC. Prof. Ogino’s research team found that *F. nucleatum* is strongly associated with CRC development and progression. They revealed a link between a low-fiber/pro-inflammatory diet and a high incidence of *F. nucleatum*-enriched colorectal cancer through a large prospective cohort study (Wang et al., 2024). In addition, they identified an inhibitory effect of *F. nucleatum* on T cell-mediated anti-tumor immunity (Borowsky et al., 2021). We built an author-based collaborative network based on the number of published papers of at least 7, visualizing 51 authors (**Figure 6**). Yu Jun’s node was the largest because she had published the most publications. The color of the same node indicates similar clusters, and it can be seen that the figure is divided into several clusters. At the same time, the collaboration between American and Chinese researchers reflects the frequent international collaboration.

**Table 2:** Top 5 authors and co-cited authors on research of *F. nucleatum* and CRC.

Rank	Authors	Publications	Co-cited authors	Citations
1	Yu Jun (China)	27	Kostic Aleksandar D (USA)	1068
2	Ogino Shuji (USA)	22	Mima K. (Japan)	813
3	Baba Hideo (Japan)	17	Rubinstein MR (USA)	759
4	Jingyuan Fang (China)	16	Yu TC (China)	480
5	Garrett Wendy S. (USA)	15	Castellarin M (Canada)	470

**Figure 7:**
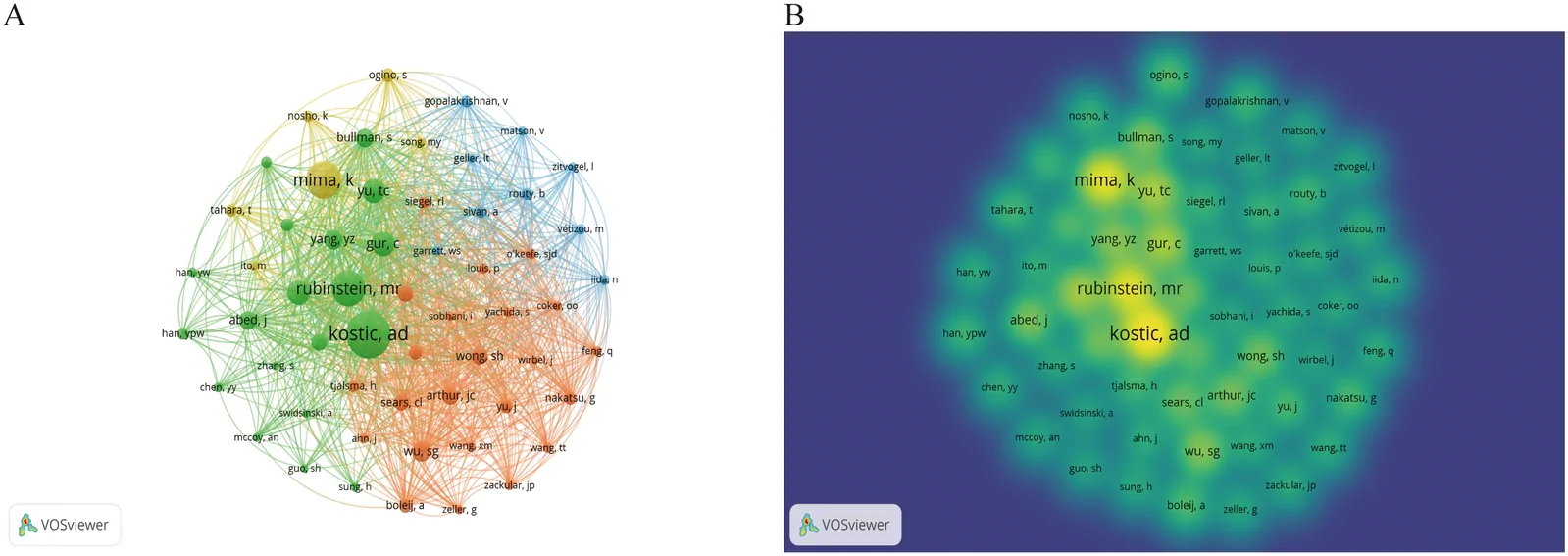
**(A)** The visualization of co-cited authors. Nodes represent cited authors; size indicates total citation count in the dataset; color denotes citation burst status; line thickness reflects co-citation frequency. **(B)** Density visualization in the visualization of co-cited authors.

Co-citation refers to two (or more) authors cited together in one or more papers. The most cited author is Kostic Aleksandar D from the United States (n =1048), followed by Mima K. from Japan (n = 813). We constructed a collaborative network based on co-cited authors based on the number of at least 130 citations, visualizing 57 co-cited authors. **Figure 7A** is the network visualization view of co-cited authors, and **Figure 7B** is the density visualization view. Kostic Aleksandar D has the largest node, indicating that his research results are widely cited (**Figure 7A**). In the density visualization, authors such as Kostic Aleksandar D, Mima, K and Rubinstein MR are the core researchers in this field (**Figure 7B**). In addition, collaboration between different high-impact authors drives progress in the field.

### 3.4 Keywords analysis

#### 3.4.1 Co-citation network of keywords

**Figure 8:**
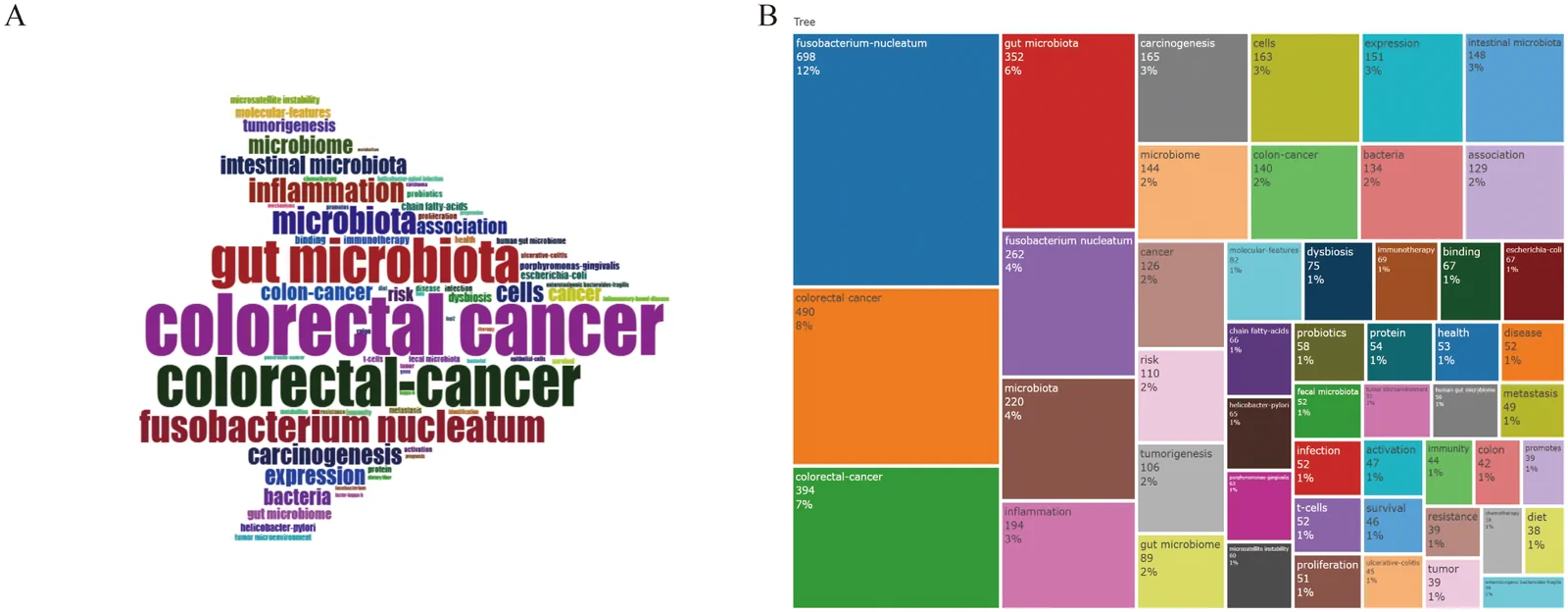
Visualization of keyword analysis. **(A)** The word cloud map of keywords in *F. nucleatum* and CRC. The word length indicates the frequency of the keyword’s appearance. **(B)** The clustering tree diagram of *F. nucleatum* and CRC. The size of the square indicates the frequency of the keyword’s appearance.

In CiteSpace, keywords play a crucial role in defining key terms and driving systematic analysis and visualization of scientific literature citation networks. With these keywords, researchers are able to identify the main research themes and key points, providing insight into research trends, hot issues, and major contributors in a particular field. As shown in **Figure 8A**, the keyword cloud map visually represents the frequency of keywords and directly reveals the key points of the research. The tags in the cloud map represent the degree of researchers’ attention to a specific topic, and the number of tags reflects the frequency of keyword usage. The higher the frequency, the more tags there are. This picture highlights the central themes of gut microbiota, microbiota, inflammation, carcinogenesis, cells, expression, intestinal microbiota microbiome.

**Figure 8B** focused on the frequency of the author’s use of keywords, among which “gut microbiota” (n = 352,9%), “microbiota” (n = 220,6%), “inflammation” (n = 194,5%), “carcinogenesis” (n = 165,4%) and “cells” (n =163,4%) was the most commonly used word.

High-frequency keywords directly reflect sustained research focuses, providing a basis for analyzing research hotspots and developmental directions.

#### 3.4.2 Keywords cluster analysis

**Figure 9:**
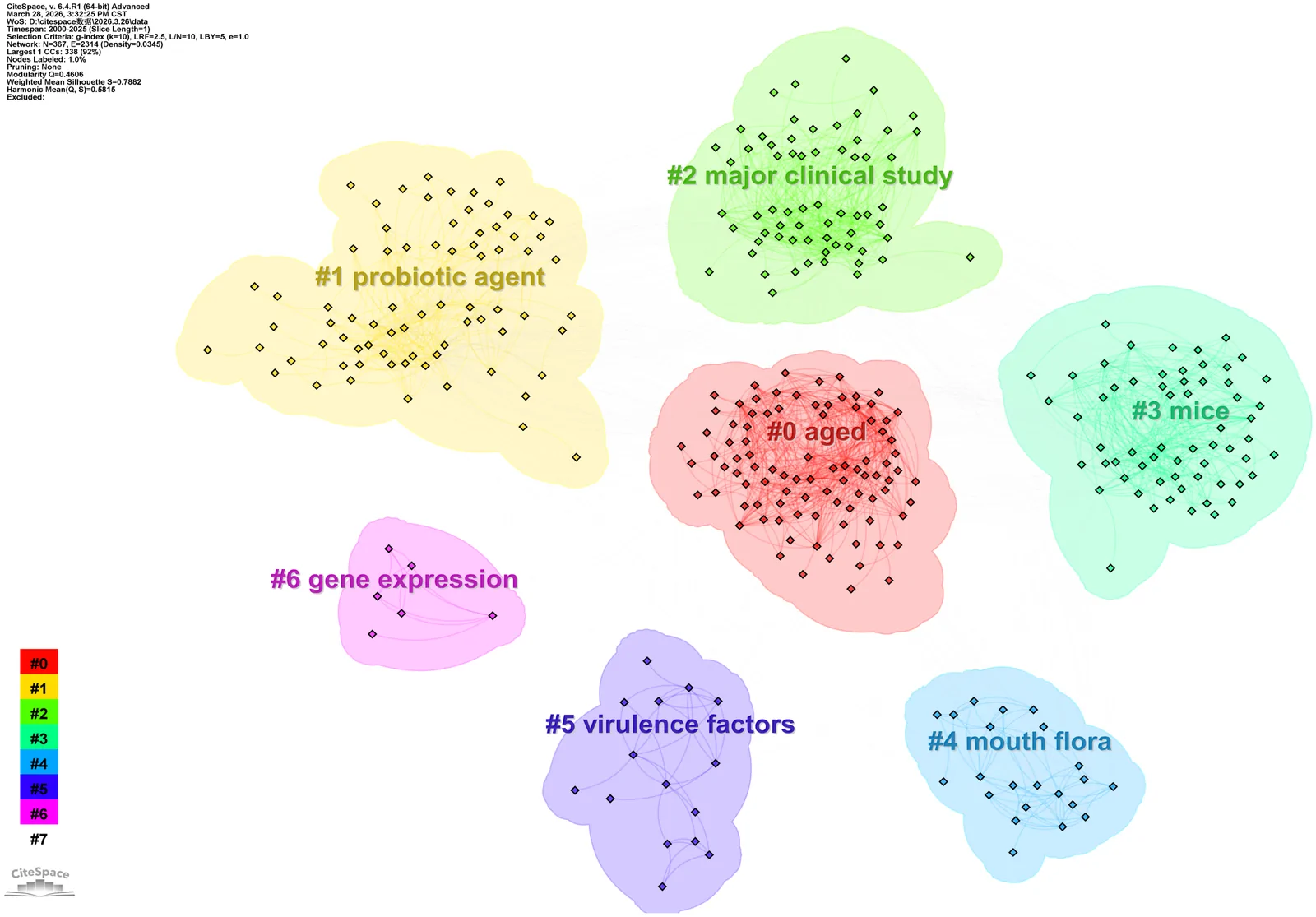
Keywords cluster analysis. Distinct colors represent different clusters. Each node corresponds to an individual keyword, and the number displayed on each node indicates the cluster to which the keyword belongs. Different node patterns also denote cluster affiliation. Clusters are assigned sequential numerical tags, with smaller tag numbers indicating clusters containing a greater number of keywords.

Cluster analysis is a machine learning method that aims to classify entities in a data set (such as literature, keywords, or samples) into different groups, often referred to as “clusters” based on their similarities or distances between them (Gao et al., 2023). In order to better reflect the research status in this field, we use CiteSpace software to generate cluster network diagram (**Figure 9**). CiteSpace will give each cluster a serial number, the smaller the serial number, the more keywords contained in the cluster. The quality of the clusters can be assessed by Q values (Modularity) and S-values (Silhouette), with Q values greater than 0.3 and S-values greater than 0.5 generally considered to mean that the clustering results are reasonable (Amrapala et al., 2023). Our result Q is 0.4606 and S is 0.7882, which proves the statistical significance of the cluster analysis. We can see in **Figure 9** that there are 7 clusters: #0 aged, #1 probiotic agent, #2 major clinical study, #3 mice, #4 mouth flora, #5 virulence factors, #6 gene expression.

The largest cluster#0 (“aged”) occupies the central position in the network, suggesting that age-related factors constitute a core focus in current studies, potentially linked to the epidemiological characteristics and risk stratification of CRC patients. “aged” cluster highlights the well-established association between older age and higher CRC risk, as well as age-related gut microbiota changes that may enhance *F. nucleatum* colonization.

Cluster #1 (“probiotic agent”) highlights the growing interest in microbiota modulation and therapeutic interventions, indicating that the role of probiotics and microbial balance in mitigating *F. nucleatum*-associated carcinogenesis has become an important research frontier. “probiotic agent” cluster reflects growing interest in using probiotics or microbial modulation to counteract *F. nucleatum*-driven carcinogenesis.

Cluster #2 (“major clinical study”) represents clinical investigations, emphasizing translational and evidence-based research, including patient cohort studies and clinical validation of microbial biomarkers. “major clinical study” cluster indicates the increasing number of large cohort studies validating *F. nucleatum* as a prognostic or predictive biomarker.

Cluster #3 (“mice”) reflects the extensive use of animal models to elucidate the mechanistic role of *F. nucleatum* in tumor initiation and progression. These studies provide experimental evidence supporting causal relationships and facilitate exploration of therapeutic targets. “mice” cluster underscores the essential role of animal models in dissecting causal mechanisms and testing therapeutic interventions.

Cluster #4 (“mouth flora”) underscores the connection between oral microbiota and CRC, supporting the hypothesis of oral-gut microbial translocation and its contribution to tumorigenesis. “mouth flora” cluster points to the oral-gut translocation hypothesis, a key concept in understanding how oral bacteria reach colorectal tumors.

Cluster #5 (“virulence factors”) focuses on the pathogenic mechanisms of *F. nucleatum*, including adhesion molecules, immune evasion, and pro-inflammatory effects, which are critical for understanding its role in promoting tumor development. “virulence factors” cluster focuses on specific bacterial molecules (e.g., FadA, Fap2) that mediate adhesion, immune evasion, and oncogenic signaling.

Cluster #6 (“gene expression”) highlights molecular-level investigations, particularly host-microbe interactions, signaling pathways, and transcriptional regulation associated with cancer progression. “gene expression” cluster captures host transcriptomic changes induced by *F. nucleatum*, linking microbial presence to cancer-related signaling pathways.

The classification and connotation of keyword clusters clearly outline mainstream research hotspots, which can further explain the evolutionary trends of this field. Overall, the clustering results demonstrated that research on *F. nucleatum* and CRC has evolved into a multidisciplinary field integrating clinical studies, mechanistic experiments, microbial ecology, and molecular biology. The identified clusters not only reveal the current hotspots but also suggest future directions, particularly in microbiome-based therapies and precision medicine.

#### 3.4.3 Keywords timeline view and time zone view

**Figure 10:**
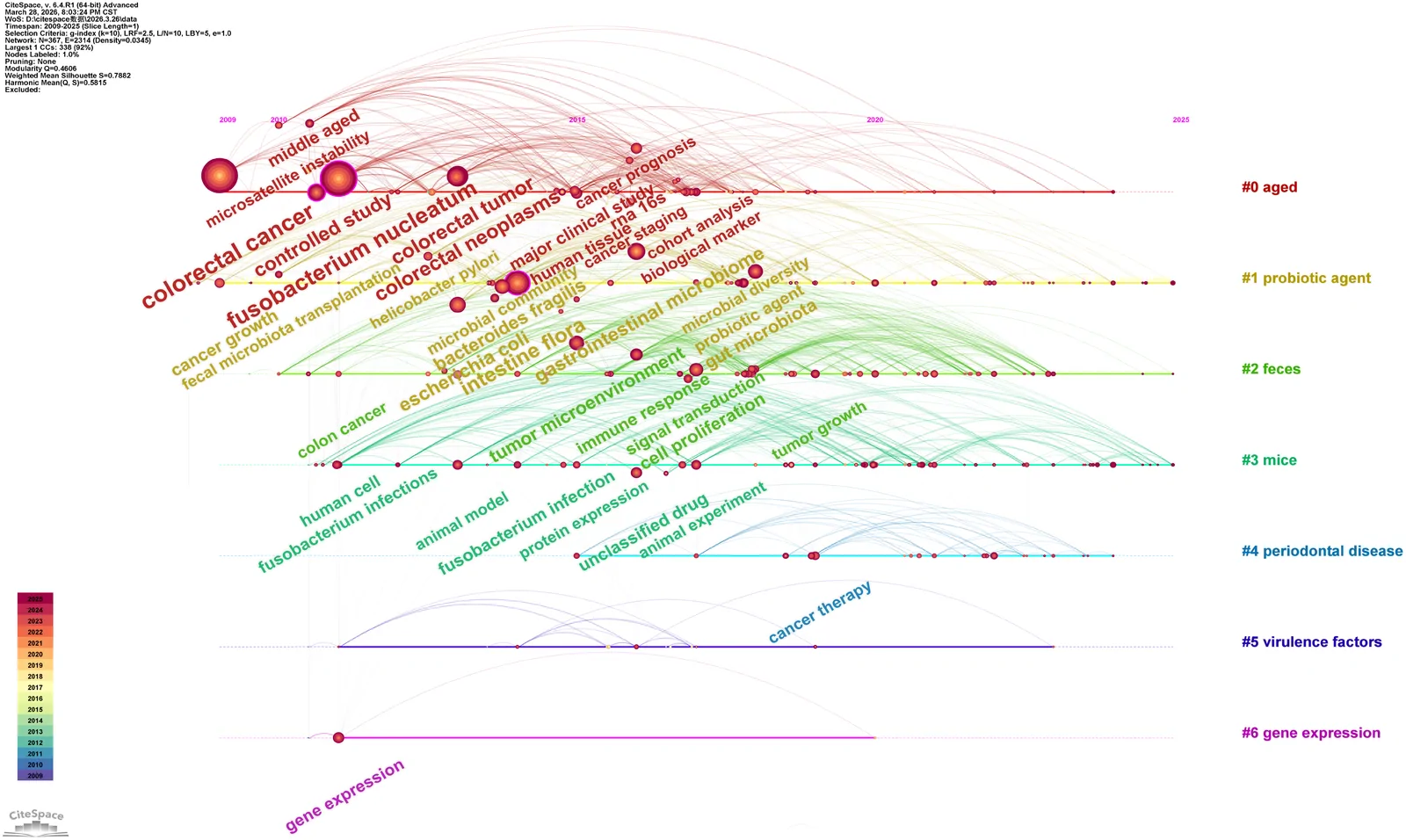
Keywords timeline view. Nodes represent keywords, sized by occurrence frequency and colored by first occurrence year. Links indicate keyword co-occurrence.

The timeline view visually displays the development trajectory of research topics over time by laying out keywords in time series. In the timeline view, each cluster (topic) is ordered and displayed on a timeline, allowing researchers to track changes in a particular topic over time (Xu et al., 2024). We generated the timeline view on the CiteSpace (**Figure 10**). The cluster tag word show a timeline at the top, from 2009 to 2025. Each year corresponds to a time point, indicating the importance of *F. nucleatum* in CRC research. Cluster #0 is the largest and longest-lasting cluster in the network, forming the backbone of the research in this field. In the early stage of the research (2009-2015), the nodes were concentrated and the nodes were large, and the frequent keywords included “colorectal cancer”, “*fusobacterium nucleatum*”, “microsatellite instability”, and “controlled study”. This indicates that the core of the early research was to establish the epidemiological connection between the enrichment of *F. nucleatum* and the occurrence of CRC, especially its correlation with specific molecular phenotypes (such as MSI status). As the research progressed, the focus gradually shifted to the complexity of the intestinal microbial ecosystem and the molecular mechanisms. In Clusters #1 and #2, “microbial community”, “gut microbiota”, “gastrointestinal microbiome”, and other specific genera (such as *Bacteroides fragilis* and *Escherichia coli*) appeared frequently. This reflects that the academic community gradually recognized that *F. nucleatum* is not an isolated carcinogen but interacts with the entire intestinal microbial network. Cluster #3 (mice) prominently showcases the prosperity of mechanism research. The emergence of keywords such as “animal model”, “tumor microenvironment”, “immune response”, “ignal transduction”, and “cell proliferation” marks that researchers extensively utilized *in vivo* and *in vitro* models to deeply analyze how *F. nucleatum* promotes the proliferation and metastasis of tumor cells by evading the host immune surveillance and regulating the immune microenvironment.

**Figure 11:**
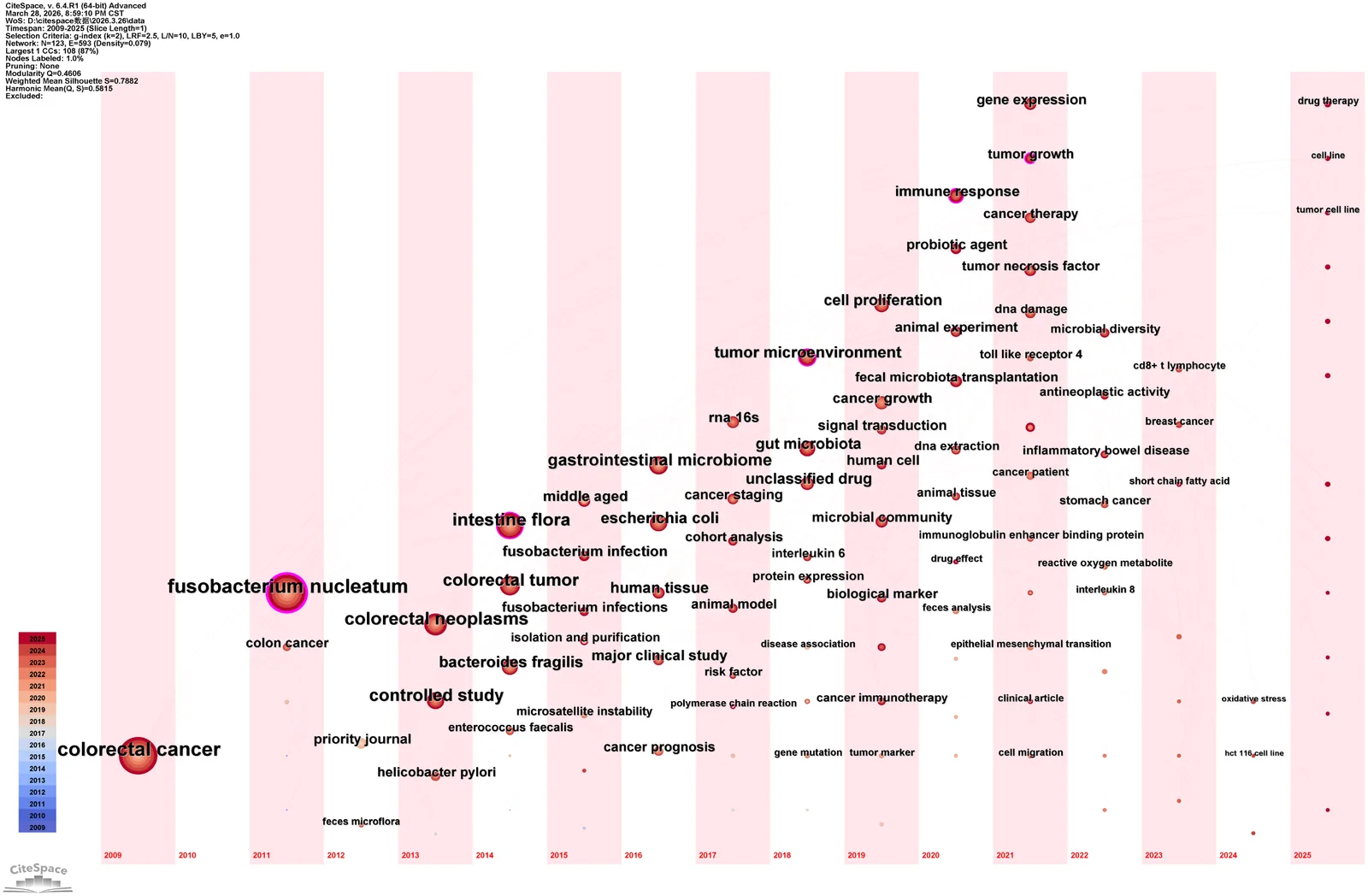
Keywords time zone view. Nodes represent keywords, sized by occurrence frequency. Clusters are color-coded, and links denote keyword co-occurrence.

The time zone view is more focused on showing reference relationships and research hotspots in different time periods. It can show the trend of reference relationships over time, segment keywords by year, and show which keywords have become key reference sources in different time periods (Li et al., 2024). We generated a time zone view through CiteSpace (**Figure 11**). The early core nodes mainly focused on establishing the basic association between “colorectal cancer” and “*fusobacterium nucleatum*”. During this stage, keywords such as “colon cancer” and “controlled study” emerged, indicating that during this period, the main approach was through clinical epidemiology and controlled studies to confirm and establish the abnormal enrichment phenomenon of this strain in intestinal tumors. As the research progressed, the focus shifted to complex micro-ecological networks and the pathogenic mechanisms within the body. The emergence of “intestine flora”, “gastrointestinal microbiome”, and “*bacteroides fragilis*” emphasized the synergistic effect of *F. nucleatum* with other intestinal microorganisms. At the same time, the dense emergence of nodes such as “tumor microenvironment”, “animal model”, and “signal transduction” marked that researchers began to extensively use *in vitro* and *in vivo* models to deeply analyze the specific molecular pathways by which *F. nucleatum* promotes cancer. Recent research frontiers have clearly tilted toward immune regulation and clinical translation. “Immune response”, “cell proliferation”, and “toll like receptor 4 (TLR4)” revealed the complex interactions between this bacterium and the host immune system. Particularly noteworthy are “cancer therapy”, “drug therapy”, and “fecal microbiota transplantation”, which have become the latest nodes, indicating that current exploration is dedicated to converting the basic mechanisms into targeted micro-ecological strategies for the treatment of colorectal cancer.

#### 3.4.4 Keyword citation burst

**Figure 12:**
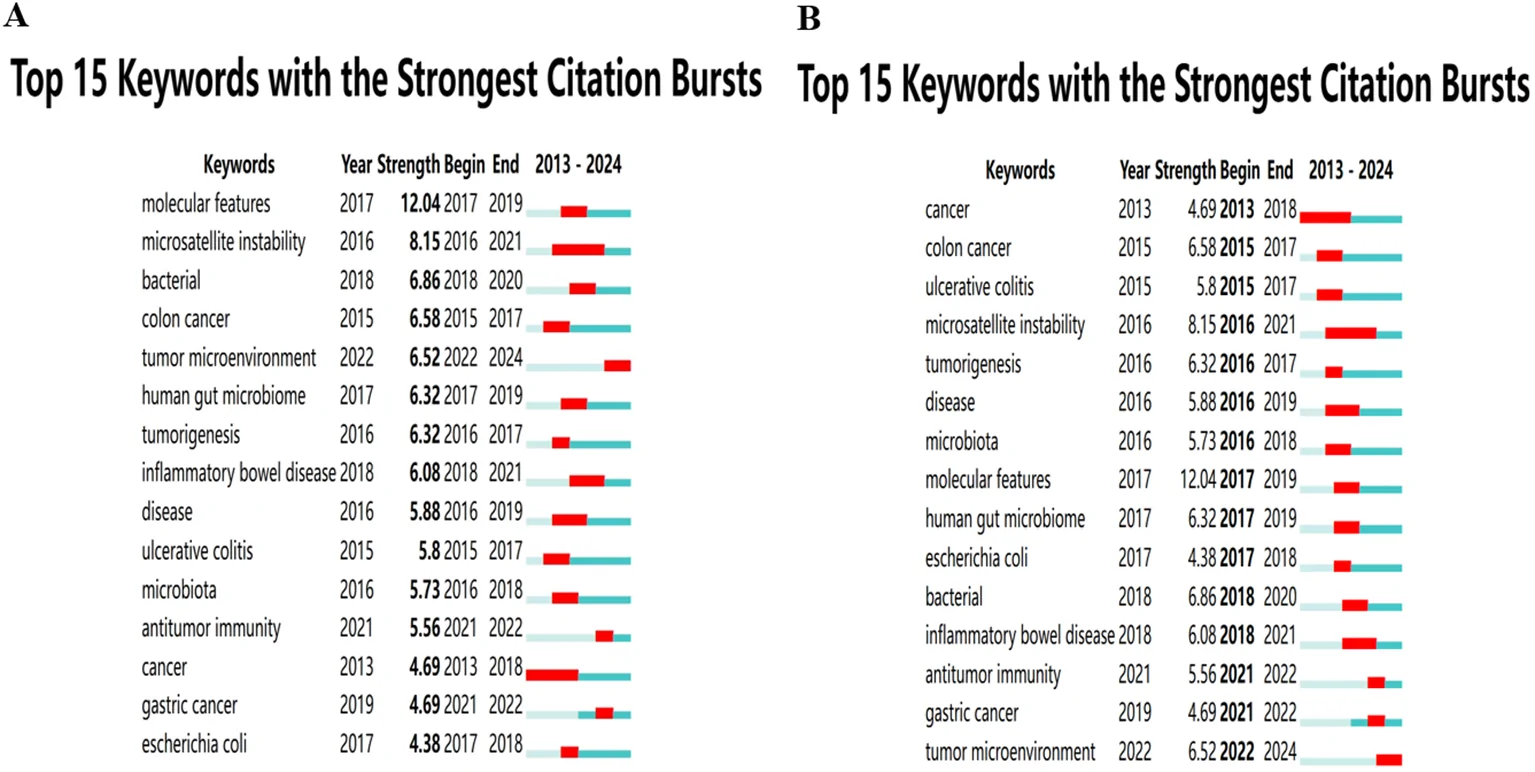
Top 15 keywords **(A)** listed in descending order by blast strength **(B)** listed in descending order by Chronological order of occurrence. The term “strength” refers to the connection intensity between two nodes, as determined by the software.

Citation burst refers to the phenomenon that the number of citations of a certain keyword increases suddenly in a specific period of time, which usually indicates the sudden increase of research heat in this field. As shown in **Figure 12**, we shows the top 15 keywords in citation burst intensity, including the time when they first appeared, burst intensity, and burst period start and end time. The blue of the burst plot refers to the time frame in which the word appeared, while the red segment highlights hot periods. **Figure 12A** is arranged in descending order of burst intensity, while **Figure 12B** is arranged chronologically based on the occurrence of the burst.

The explosive intensity of the keyword “molecular features” is the highest, and the explosive intensity from 2017 to 2019 is 12.04. The outburst intensity of the keyword “microsatellite instability” is much less than the former, 8.15 (period: 2016-2021).The third keyword “bacterial” performed with an outbreak intensity of 6.86 from 2016 to 2021. Other keywords included colon cancer (strength: 6.58, period: 2015-2017), tumor microenvironment (6.52, 2022-2024), human gut microbiome (6.32, 2017-2019), tumorigenesis (6.32, 2016-2017), inflammatory bowel disease (6.08, 2018-2021), disease (5.88, 2016-2019), ulcerative colitis (5.8, 2015-2017), microbiota (5.73, 2016-2018), antitumor immunity (5.56, 2021-2022), cancer (4.69, 2013-2018), gastric cancer (4.69, 2021-2022) and escherichia coli (4.38, 2017-2018). The proliferation of the above keywords reflects the current academic attention and attention in the fields of cancer research, gut microbiome, disease mechanism and immune response.

### 3.5 Co-cited journals

There are a total of 151 cited journals related to *F. nucleatum* and CRC. We counted the top 10 cited journals (**Table 3**). The journal Gut was the most cited (n=971), followed by Cell Host & Microbe journals (n=945) and PLoS One journals (n=873). The citations of the top ten cited journals all reached more than 600. In addition, the JCR partitions of all journals are Q1 zones, showing overall excellent quality. The highest IF score among all journals was Nature (IF = 48.5), followed by Science (IF = 45.8) and Cell (IF = 42.5), which generated a visual network of cited journals (**Supplementary Figure 1**). The larger nodes in the graph, such as “Nature”, “Science”, “Gut”, “Cell” show that these journals have a high impact in the field.

**Table 3:** Top 10 co-cited journals on research of *F. nucleatum* and CRC.

Rank	Co-cited journal	Co-citations	IF (2025)	JCR (2025)
1	Gut	971	26.2	Q1
2	Cell Host & Microbe	945	18.7	Q1
3	PLoS One	873	2.6	Q1
4	Science	869	45.8	Q1
5	Gastroenterology	866	25.9	Q1
6	Cell	800	42.5	Q1
7	Nature	748	48.5	Q1
8	Nature Communications	692	15.7	Q1
9	P Natl Acad SCI USA	670	9.1	Q1
10	Scientific Reports	663	3.9	Q1

The high co-citation counts for journals such as Gut, Cell Host & Microbe, and Gastroenterology reflect their role as primary outlets for mechanistic and translational studies on gut microbiota-cancer interactions. These journals consistently publish high-impact original research and authoritative reviews on *F. nucleatum* and CRC, shaping the field’s intellectual agenda. Furthermore, the inclusion of open-access journals (PLoS One, Scientific Reports) suggests that the field benefits from wide dissemination of incremental findings and methodologically sound studies. Thus, the co-citation landscape not only identifies the most influential journals but also reveals the multidisciplinary nature of this research area-spanning clinical gastroenterology, basic microbiology, immunology, and high-profile general science.

### 3.6 Citation burst references

**Figure 13:**
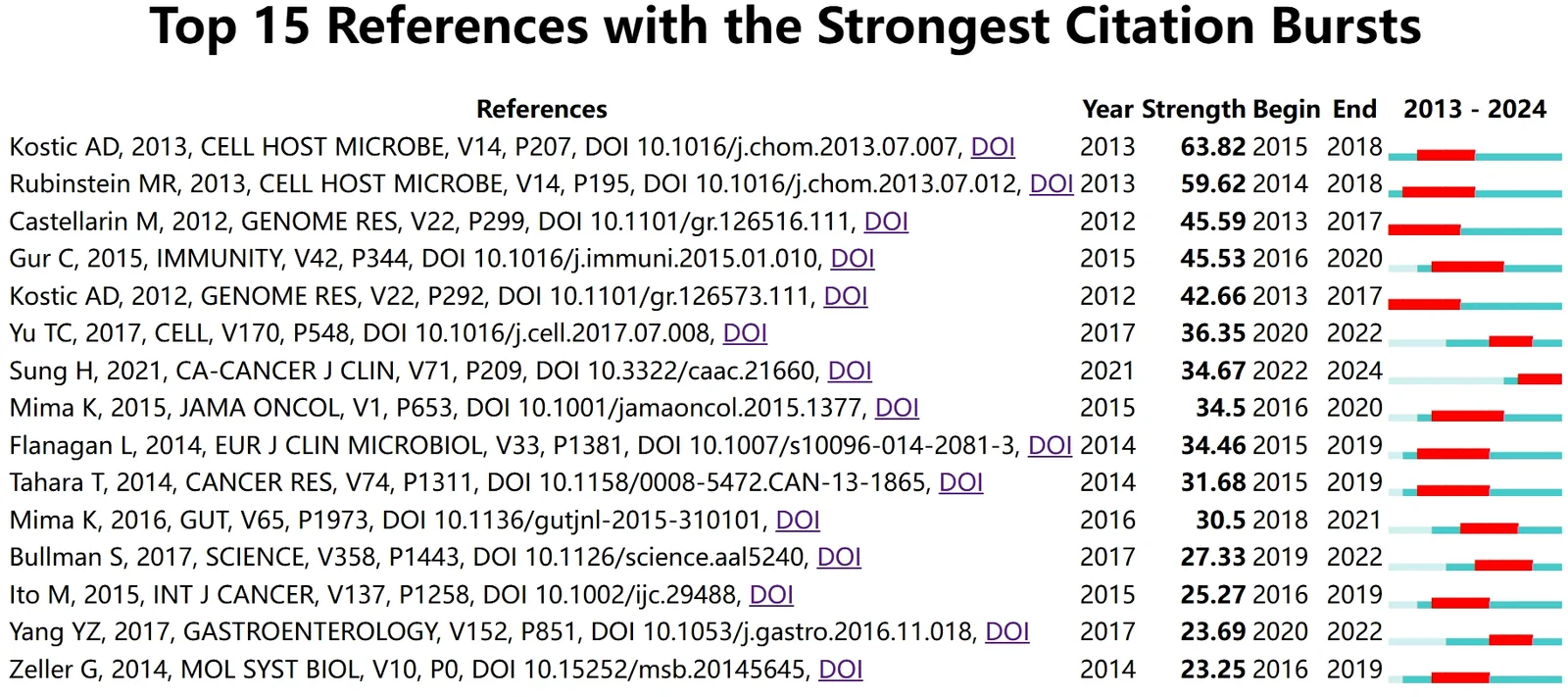
Top 15 references listed in descending order by blast strength.

In this study, we used CiteSpace to identify top15 references with strong citation bursts. As shown in **Figure 13**, the red bars represent strong citation bursts. The references with outbreaks range from 2012-2021, and the range of outbreak strength is 23.25-63.82. We also listed the titles, journals, IFs, JCRs, and DOI links for these 15 references (**Supplementary Table 1**). The one with the highest IF is entitled Global Cancer Statistics 2020: GLOBOCAN Estimates of Incidence and Mortality Worldwide for 36 Cancers in 185 Countries, published in CA: A Cancer Journal for Clinicians (IF = 232.4).

## 4 Discussion

### 4.1 General information

The current research field does not focus on the bibliometrics between *F. nucleatum* and CRC. Using bibliometric methods, this study comprehensively analyzed the research related to *F. nucleatum* and CRC, pinpointed the research hotspots and trends, and outlined the key directions for subsequent research.

#### 4.1.1 Discussion on the annual publication volume

This study obtained 2010 publications on *F. nucleatum* and CRC by searching WOSCC and Scopus from 2000 to 2025. The maturation of next-generation metagenomic sequencing enabled the first unbiased identification of *F. nucleatum* enrichment in CRC tissues, which established the core research question and opened up this field. The development trajectory of this research field can be roughly divided into three distinct stages. The period of rapid growth was from 2017 to 2021. The number of published papers increased sharply from 88 in 2017 to 258 in 2021. The top ten countries in terms of publication volume are located in Asia, Europe and North America. Over the course of the study, China was the highest contributor to the relationship between *F. nucleatum* and CRC, with the highest number of publications, followed by the United States and Italy. It was worth noting that seven of the top ten institutions were located in the United States and three in China. The active and effective collaboration between countries and institutions has facilitated research on the relationship between *F. nucleatum* and CRC.

#### 4.1.2 Discussion on the influence of the author

In terms of authors, Prof. Yu Jun from The Chinese University of Hong Kong, China, who has published the most articles, has long been engaged in basic and translational research on the molecular mechanisms of digestive tumors, intestinal micro-ecology, fatty liver, tumor molecular markers, and anti-tumor therapeutic targets. Her research team found that the abundance of *F. nucleatum* may be a biomarker for predicting the effectiveness of anti-PD-1 therapy for MSS CRC (Wang et al., 2025). Prof. Shuji Ogino of the Harvard T.H. Chan School of Public Health, U.S.A., as the second most published author, has also made significant contributions to the study of the relationship between *F. nucleatum* and CRC. He noted that the presence of *F. nucleatum* is significantly correlated with different molecular subtypes of CRC (especially the high-inflammatory-responsive subtype), which may influence the biological behavior of the cancer (Mima et al., 2016). It is these high-impact investigators who are driving the knowledge of the relationship between *F. nucleatum* and CRC, providing new ideas and strategies for the immunotherapy of CRC patients, as well as new directions in anti-tumor immunology research.

#### 4.1.3 Discussion of keyword trends

Keywords provided quick access to the distribution and evolution of hotspots in a research area. By analyzing high-frequency keywords, hot issues within a research field can be quickly identified. These high-frequency keywords reflected topics that continue to receive attention within the field. The results of keyword clustering indicate that “aged”, “probiotic agent”, “major clinical study”, “mice”, “mouth flora”, “virulence factors”, “gene expression” and other terms reflect past research trends. The keyword timeline and time zone views reveal sustained growth in research focused on immunotherapy, host gene regulation, and gut microbiota dysbiosis.

#### 4.1.4 Discussion on journal impact

In terms of the distribution of cited journals, the journal Gut was the most cited (n=971), followed by the journal Cell Host & Microbe (n=945) and the journal PLoS One (n=873). The top 10 cited journals all had more than 600 citations. In addition, all journals were Q1 partitioned (JCR partitioning), demonstrating excellent quality and science. Among all journals, Nature (IF = 48.5) has the highest IF score, followed by Science (IF = 45.8) and Cell (IF = 42.5). Analyzing the distribution of sources can help to identify the core journals that publish research on *F. nucleatum* and CRC, which is important for scholars to establish their research results.

### 4.2 Molecular mechanism of *F. nucleatum* promoting CRC

*F. nucleatum* is a common oral anaerobic Gram-negative bacterium whose abundance in CRC tissues was significantly higher than in normal intestinal mucosa or paracancerous tissues (Castellarin et al., 2012). In 2012, macro-genomics studies reported for the first time the enrichment of *F. nucleatum* in CRC tissues, which was detected at a rate of up to 30-50% and correlated with tumor stage and poor prognosis (Kostic et al., 2013). A study of a global multicenter cohort further demonstrated that *F. nucleatum* loadings in stool samples from CRC patients were 2–3 times higher than in healthy populations, suggesting its potential use as a non-invasive biomarker (Yachida et al., 2019).

The “molecular features” (**Figure 12**) burst coincides with structural characterization of FadA. *F. nucleatum* binds to host cell E-cadherin through the surface adhesin FadA, which activates the β-catenin signaling pathway, induces the expression of oncogenes, and inhibits apoptosis (Rubinstein et al., 2019). FadA also disrupts intercellular junctions through activation of endocytosis of the E-cadherin/β-catenin complex, and promotes Epithelial mesenchymal transition (EMT) (Galasso et al., 2025).

Fap2 enhances bacterial colonization of CRC cells by binding to Gal-GalNAc polysaccharides on the surface of tumor cells (Abed et al., 2016). *F. nucleatum* recruits myeloid-derived suppressor cells (MDSCs) and inhibits cytotoxic CD8^+^T-cell activity via the Fap2 lectin, fostering an immunosuppressive tumor microenvironment (Bullman et al., 2017; Gur et al., 2019). Fap2 also has an immunomodulatory function that can block the killing activity of natural killer cells (NK cells) on tumor cells by binding to the inhibitory receptor TIGIT on the surface of NK cells (Pignatelli et al., 2023). Animal models showed that *F. nucleatum* significantly increased the number and volume of intestinal adenomas in Apc^Min/+^ mice, and the effect was dependent on FadA expression (Brennan et al., 2021). Recently, RadD, a new *F. nucleatum* adhesin with pro-oncogenic effects, was found to activate the PI3K-AKT-NF-κB-MMP9 pro-oncogenic signaling pathway by directly binding to the colorectal cancer cell surface receptor CD147, ultimately promoting tumor growth and metastasis (Zhang et al., 2024). The major adhesins of F. nucleatum and their functional roles in CRC are summarized in **Table 4**.

Regarding the “cancer immunotherapy” and “tumor microenvironment” mentioned in **Figure 11**, we have focused our attention on the field of immunology. *F. nucleatum* activates NF-κB through the TLR4/MyD88 signaling pathway and promotes the release of pro-inflammatory factors (IL-6, IL-8, TNF-α) to create a chronic inflammatory environment (Hsueh et al., 2022). Its metabolite butyrate down-regulates the expression of *F. nucleatum* adhesion-associated outer membrane proteins (e.g., RadD, FomA, and FadA), thereby reducing its colonization and invasion on colorectal cancer cells (Chen et al., 2023). In addition, *F. nucleatum* releases outer membrane vesicles, inducing the production of pro-inflammatory cytokines and initiating inflammation (Pignatelli et al., 2023). In addition, *F. nucleatum* binds to T cell immune checkpoint receptor TIGIT via Fap2 protein, inhibits CD8^+^ T cell activity, and induces the expansion of regulatory T cells to form an immunosuppressive microenvironment (Jiang et al., 2023). In patients with metastatic CRC, *F. nucleatum* promotes CRC metastasis through miR-1322/CCL20 axis and M2 polarization (Xu et al., 2021).

*F. nucleatum* plays a significant role in metabolism*. F. nucleatum* can influence metabolic reprogramming and tumor stem cell maintenance, which can redirect metabolism to increase formic acid secretion and cancer glutamine metabolism.Among them, formic acid drives colorectal cancer tumor invasion by triggering AhR signaling, while increasing cancer stemness (Ternes et al., 2022). Notably, cancer cells from patients with relatively high *F. nucleatum* levels were characterized by cancer stem cells (CSCs) (Huang et al., 2020). *F. nucleatum* can promote self-renewal of colorectal cancer stem-like cells (CCSC) by manipulating cellular lipid accumulation and enable non-CCSC to acquire CCSC characteristics (Liu et al., 2022a).

**Table 4:** Summary of major *F. nucleatum* adhesins and their roles in CRC.

Adhesin	Receptor/binding target	Core mechanism	CRC-related effects	Key references
FadA	E-cadherin (host cell)	Activates β-catenin signaling; induces oncogene expression; disrupts intercellular junctions; promotes EMT	Tumor initiation, proliferation, invasion	(Rubinstein et al., 2019; Galasso et al., 2025)
Fap2	Gal-GalNAc (tumor surface polysaccharide); TIGIT (NK and T cells)	Mediates bacterial colonization via Gal-GalNAc binding; inhibits NK cells cytotoxicity and CD8^+^ T-cell activity via TIGIT engagement; recruits MDSCs	Tumor colonization, immunosuppression, immune evasion	(Gur et al., 2015, 2019; Abed et al., 2016)
RadD	CD147 (CRC cell surface receptor)	Activates PI3K-AKT-NF-κB-MMP9 pro-oncogenic signaling pathway	Tumor growth, metastasis	(Zhang et al., 2024)

### 4.3 Research hotspots and emerging frontiers

The keyword co-occurrence and burst analysis revealed distinct research hotspots and evolving frontiers in *F. nucleatum*-CRC studies. Terms such as ‘colorectal cancer’ (centrality=0.55) and ‘*Fusobacterium nucleatum*’ (centrality=0.37) exhibit the highest betweenness centrality, underscoring their foundational role as core connecting points in the research network. Notably, the keyword “tumor microenvironment” exhibited a strong citation burst (strength=6.52, 2022–2024), reflecting intensified interest in how *F. nucleatum* reshapes immune landscapes to promote carcinogenesis. Recent studies demonstrate that *F. nucleatum* recruits myeloid-derived suppressor cells (MDSCs) and inhibits cytotoxic T-cell activity, creating an immunosuppressive niche conducive to tumor progression (Zhang et al., 2024). The NF-κB signaling pathway may be the key pathway linking microbial dysbiosis with chronic inflammation and immune escape (Wong and Yu, 2023).

The temporal evolution of keywords further highlights shifting priorities. Early research (2013–2017) focused on molecular mechanisms, as evidenced by bursts in “molecular features” (strength=12.04) and “microsatellite instability” (strength=8.15). These studies established *F. nucleatum* as a biomarker for CRC subtypes, particularly MSS tumors resistant to conventional therapies (Mima et al., 2016). Post-2020, the field pivoted toward translational applications, with surges in “antitumor immunity” (strength=5.56) and “dysbiosis” (strength=6.32). For instance, Yu Jun’s landmark work revealed that *F. nucleatum*-enriched tumors exhibit enhanced PD-1 blockade responsiveness when combined with butyrate supplementation, offering a novel combinatorial strategy for MSS-CRC immunotherapy (Wang et al., 2025).

The convergence of keyword bursts in “immunotherapy” and “dysbiosis” (both post-2020) suggests that the field is transitioning from mechanistic discovery toward clinical application. This bibliometric observation aligns with ongoing phase I/II trials testing microbiota modulation in CRC patients, though causal validation remains pending.

### 4.4 Limitations

While this bibliometric analysis provides a comprehensive overview, several limitations warrant consideration. First, the research data are derived from the WOS databases and Scopus, which may have excluded relevant studies from PubMed, Medline, Embase, etc. We acknowledge that the exclusion of additional databases may introduce a degree of selection bias. Future studies could incorporate more databases to perform cross-validation. Second, keyword clustering in CiteSpace depends on algorithmic parameters (e.g., LRF = 2.5, g-index=2), which may introduce subjectivity in thematic boundaries. Third, restricting to English-language publications may introduce language bias, particularly given China’s leading output in this field. However, most high-impact Chinese studies on *F. nucleatum* and CRC are published in English, and our bibliometric tools require standardized data from WOSCC/Scopus. Future studies could incorporate Chinese databases (e.g., CNKI) for cross-validation. In particular, it should be emphasized that bibliometric analyses are inherently descriptive and cannot establish causal relationships or quantify the strength of biological mechanisms. Bibliometric analysis is a descriptive tool that can identify trends, collaborations, and research hotspots, but it cannot establish causal relationships between variables (e.g., between *F. nucleatum* and CRC outcomes) nor directly generate clinical recommendations.

### 4.5 Future perspectives

The sharply declining cost of sequencing technologies allowed large-cohort clinical validation; sustained funding investment in gut microbiome and tumor microbiome research (e.g., the NIH Human Microbiome Project in the US and specialized grants from the National Natural Science Foundation of China) expanded research teams; and a shift in research priorities from correlation verification to mechanistic dissection, driven by clinical demands for solving chemotherapy resistance in CRC.

This study delineates the intellectual architecture of *F. nucleatum*-CRC research, identifying three pivotal trends: (1) the transition from mechanistic exploration to therapeutic innovation, (2) the growing emphasis on microbiota-immune crosstalk, and (3) the rise of multidisciplinary approaches integrating genomics, metabolomics, and immunology. Looking ahead, three directions hold promise (1) Microbiota-Targeted Therapies: Clinical trials testing *F. nucleatum*-specific antibiotics (e.g., metronidazole) or probiotics to restore microbial balance in CRC patients (Wong and Yu, 2019). (2) Biomarker Development: Validating *F. nucleatum* load as a non-invasive diagnostic tool for early-stage CRC and chemotherapy resistance monitoring (Wirbel et al., 2019). (3) Multi-Omics Integration: Combining metagenomics, single-cell sequencing, and spatial transcriptomics to unravel host-microbe interactions at the tumor margin (Wu et al., 2023). By addressing these priorities, researchers may generate hypotheses that could eventually lead to improved CRC outcomes, but direct clinical translation requires confirmatory studies.

In order to link our bibliometric findings with future research directions and facilitate the transformation, we propose the following suggestions: We recommend prioritizing translational research that bridges the gap between mechanistic discoveries and clinical applications, as the field has now entered the mature high-output phase focused on therapeutic innovation. We call for enhanced international collaboration between China, the United States, and European institutions, particularly in the areas of multi-center clinical trials and standardization of *F. nucleatum* detection methods. We suggest establishing a global research network led by the most productive institutions and authors to share data, samples, and technical resources, accelerating progress in the field.

## 5 Conclusion

By using VOSviewer, CiteSpace and Bibliometrix to conduct a visual analysis of countries, institutions, authors, keywords, journals and references, this bibliometric analysis depicted the evolving knowledge landscape of *F. nucleatum* and CRC research, revealing three changing trends. First, the field has transitioned from foundational studies on molecular mechanisms (e.g., FadA-mediated β-catenin activation) to translational innovations targeting the tumor microenvironment and immunotherapy resistance. Second, global collaboration networks, led by the U.S. and China, have accelerated discoveries in microbiota-host interactions, though regional disparities in resource allocation persist. Third, emerging frontiers such as “tumor microenvironment” (burst strength=6.52) and “antitumor immunity” (burst strength=5.56) underscore the growing emphasis on microbial modulation of immune checkpoints. Ultimately, prioritizing *F. nucleatum*-specific therapies validating microbial biomarkers for early diagnosis, and integrating multi-omics platforms will be critical to translating microbiome insights into clinical advances and potential applications. This study provides a systematic perspective and valuable insights for advancing CRC therapeutics through interdisciplinary collaboration and microbiota-centric strategies.

For researchers, the identified collaboration networks and keyword clusters can help identify potential partners, core journals, and emerging topics for future investigations. For funding agencies and policy makers, the visualized trends may inform priority-setting and resource allocation. For clinical investigators, the hotspots in ‘tumor microenvironment’ and ‘antitumor immunity’ highlight areas where translational efforts (e.g., biomarker validation, combination therapy trials) may be most promising. However, these bibliometric insights should be viewed as hypothesis-generating tools; they can suggest directions but cannot replace mechanistic studies or clinical trials.

## Data Availability

The original contributions presented in the study are included in the article/**Supplementary Material**. Further inquiries can be directed to the corresponding author.
