# Supplementary material for: Global research trends in Fusobacterium nucleatum and colorectal cancer: a bibliometric and visual analysis
Source: Front Cell Infect Microbiol. 2026 Sep 3;16:1851307. doi: 10.3389/fcimb.2026.1851307 (PMC13581885; doi:10.3389/fcimb.2026.1851307)
Supplement: Supplementary file 1 [file Table1.docx]

**Figure S1** Graph of cited journals

| Rank | Title | Journal | IF (2025) | JCR (2025) | DOI |
| --- | --- | --- | --- | --- | --- |
| 1 | Fusobacterium nucleatum Potentiates Intestinal Tumorigenesis and Modulates the Tumor-Immune Microenvironment | Cell Host & Microbe | 18.7 | Q1 | 10.1016/j.bios.2021.113932 |
| 2 | Fusobacterium nucleatum Promotes Colorectal Carcinogenesis by Modulating E-Cadherin/β-Catenin Signaling via its FadA Adhesin | Cell Host & Microbe | 18.7 | Q1 | 10.1016/j.chom.2013.07.012 |
| 3 | Fusobacterium nucleatum infection is prevalent in human colorectal carcinoma | Genome Research | 5.5 | Q1 | 10.1101/gr.126516.111 |
| 4 | Binding of the Fap2 Protein of Fusobacterium nucleatum to Human Inhibitory Receptor TIGIT Protects Tumors from Immune Cell Attack | Immunity | 26.3 | Q1 | 10.1016/j.immuni.2015.01.010 |
| 5 | Genomic analysis identifies association of Fusobacterium with colorectal carcinoma | Genome Research | 5.5 | Q1 | 10.1101/gr.126573.111 |
| 6 | Fusobacterium nucleatum Promotes Chemoresistance to Colorectal Cancer by Modulating Autophagy | Cell | 42.5 | Q1 | 10.1016/j.cell.2017.07.008 |
| 7 | Global Cancer Statistics 2020: GLOBOCAN Estimates of Incidence and Mortality Worldwide for 36 Cancers in 185 Countries | CA: A Cancer Journal for Clinicians | 232.4 | Q1 | 10.3322/caac.21660 |
| 8 | Fusobacterium nucleatum and T Cells in Colorectal Carcinoma | JAMA Oncology | 20.1 | Q1 | 10.1001/jamaoncol.2015.1377 |
| 9 | Fusobacterium nucleatum associates with stages of colorectal neoplasia development, colorectal cancer and disease outcome | Gut | 25.8 | Q1 | 10.1136/gutjnl-2015-310101 |
| 10 | Fusobacterium in Colonic Flora and Molecular Features of Colorectal Carcinoma | Cancer  Research | 16.6 | Q1 | 10.1158/0008-5472.CAN-13-1865 |
| 11 | Fusobacterium nucleatum in colorectal carcinoma tissue and patient prognosis | Gut | 25.8 | Q1 | 10.1136/gutjnl-2015-310101 |
| 12 | Analysis of Fusobacterium persistence and antibiotic response in colorectal cancer | Science | 45.8· | Q1 | 10.1126/science.aal5240 |
| 13 | Association of Fusobacterium nucleatum with clinical and molecular features in colorectal serrated pathway | International journal of cancer | 4.7 | Q1 | 10.1002/ijc.29488 |
| 14 | Fusobacterium nucleatum Increases Proliferation of Colorectal Cancer Cells and Tumor Development in Mice by Activating Toll-Like Receptor 4 Signaling to Nuclear Factor−κB, and Up-regulating Expression of MicroRNA-21 | Gastroenterology | 25.1 | Q1 | 10.1053/j.gastro.2016.11.018 |
| 15 | Potential of fecal microbiota for early‐stage detection of colorectal cancer | Molecular Systems Biology | 7.7 | Q1 | 10.15252/msb.20145645 |

**Table S1** Top15 cited references’ title, journal, IF, JCR and DOI
